# Cross-cultural differences in foreign language learning strategy preferences among Hungarian, Chinese and Mongolian University students

**DOI:** 10.1016/j.heliyon.2021.e06505

**Published:** 2021-03-16

**Authors:** Anita Habók, Yunjun Kong, Jargaltuya Ragchaa, Andrea Magyar

**Affiliations:** a Institute of Education, University of Szeged, Hungary; b Doctoral School of Education, University of Szeged, Hungary; c Center for Research on Learning and Instruction, University of Szeged, Hungary

**Keywords:** Cultural differences, Language learning strategy, Foreign language learning, University students, Cross cultural comparison

## Abstract

The present study explores English as a foreign language (EFL) learning strategies used in Hungarian, Chinese, and Mongolian university students with different cultural and linguistic backgrounds. A total of 519 university students participated in the survey from the three different countries. The Strategy Inventory for Language Learning (SILL), developed by Rebecca Oxford (2003), was administered to explore cross-cultural differences in strategy use in the study. To explain cultural divergences, we employed Hofstede's 6-D model of cultural values. The study identified a number of cross-cultural similarities and differences in strategy use among these three groups. All the subsamples similarly preferred the use of metacognitive learning strategies; however, there were some significant differences among the countries. A significant difference was observed in cognitive language learning strategy usage for the Hungarian subsample compared to the Mongolian subsample. With regard to the affective field, we noticed that the Mongolian and Chinese students employed affective strategies significantly more frequently. The Hungarian students rated the use of affective strategies the lowest by comparison. These differences may be partly linked to the cultural traditions of the participating countries. Our findings also suggest that although students' cultural background is a significant factor, linguistic and educational background and teaching traditions are also crucial.

## 1 Introduction

Foreign language (FL) learners employ a number of different techniques, methods or strategies to aid them in making language learning more successful and self-directed. Language learning strategies (LLSs) are recognized as significant contributing factors in successful language learning. LLSs have been defined in several ways (Oxford, 2017), with one of the most widely accepted definitions of LLSs being “specific actions taken by the learner to make learning easier, faster, more enjoyable, more self-directed, more effective and more transferable to a new situation” (Oxford, 1990, p. 8). Thus, FL learners who can adequately select and use language learning strategies and have the ability to monitor their own strategy use through the entire process of their learning become more self-regulated and more successful language learners (Anderson, 2003).

Research has shown that several factors have a significant impact on learners' strategy use, and many studies emphasize the role of cultural or national divergences. The notion of culture is defined as “the collective programming of the mind that distinguishes the members of one group or category of people from others” (Hofstede, 2011, p. 3). The majority of studies have focused on investigating different nations’ English language learning strategies (EFL), e.g. Chinese (Deneme, 2008; Hu, 2010), Hungarian (Doró and Habók, 2013; Habók & Magyar, 2018a, 2020), Greek (Kambakis-Vougiouklis and Mamoukari, 2016), Singaporean (Gu et al., 2005; Wharton, 2000) and Taiwanese (Wu, 2008). Relatively little research has focused on mapping cross-cultural divergences in FL strategy use among different nations (e.g. Banasiak-Ryba, 2010; Deneme, 2010; Hong-Nam and Leavell, 2007; Jiang and Wu, 2016; Oxford, 1996). The overall consensus of these studies is that language learning strategy use is shaped by the different cultural and educational backgrounds of the learners (Oxford, 1996). Less is known, however, about how English language learners of different cultural and educational backgrounds actually use language learning strategies.

The main aim of this study is to shed more light on this area of research by exploring the similarities and differences in FL strategy use among university students of three different cultural, linguistic and educational backgrounds. We are attempting to discover the main characteristics of university students’ language learning strategies in one European (Hungarian) and two Asian (Chinese and Mongolian) contexts.

## 2 Literature review

### 2.1 Foreign language learning strategies

Since the mid-1970s, learning strategy use has been one of the most frequently studied area of FL learning (Alhaysony, 2017; Charoento, 2016; Chen, 2014; Dawadi, 2017; Doró and Habók, 2013; Habók & Magyar, 2018a, 2020; Jiang and Cohen, 2012; Oxford, 1990, 2003, 2017; Wu, 2008; Zhang and Xiao, 2006). Several definitions and classifications have been presented, the most influential being Oxford's taxonomy, which groups strategies in two main categories: direct and indirect strategies (Oxford, 2017). The direct strategies contain three strategy domains: (1) memory strategies, which contribute to the reception, storage and recall of information; (2) cognitive strategies, which make it possible to work with information in which mental processes play a major role; and (3) compensation strategies, which aid in processing or transmitting information even when language barriers arise or there are gaps in communication. Oxford's indirect strategies also comprise of three strategy types: (1) metacognitive strategies, which focus on learning planning, achievement of learning goals, and monitoring and evaluation of activities; (2) affective strategies, which deal with language learning-related emotions; and (3) social strategies, which are linked to communication-based language learning, in which the role of highly proficient speakers also occurs (Oxford, 1990). Based on this classification system, Oxford developed the *Strategy Inventory for Language Learning* (SILL, Oxford, 1990, 2003), which, even today, is the most frequently applied questionnaire used to study learning strategies. Recently, Oxford reconsidered her taxonomy and constructed a new model with four strategy categories on the basis of the sociocultural theory of self-regulated learning: the cognitive, social, motivational and affective strategies, which are guided by metacognitive, metasocial, metamotivational and meta-affective strategies, respectively (Oxford, 2017). However, she has not elaborated on this strategy classification; therefore we relied on her original taxonomy.

### 2.2 Dimensions of culture

FL learning is situated in a particular cultural context and may be influenced by the cultural values of the language learner. For instance, in a culture where personal competition is stressed, students tend to use strategies which involve working individually instead of social strategies that promote cooperation with their peers (Chamot, 2004). However, Oxford (1996) has pointed out that it can be harmful to restrict particular types of strategy use for a particular society. Strategy use is also greatly dependent on other sociocultural factors, e.g. linguistic or pedagogical background (Oxford and Gkonou, 2018).

In the second half of the twentieth century, Hofstede (2001) identified a way to describe dimensions of culture. He established a 6-D model of cultural values, which measured differences among cultures and calculated indices in six distinct dimensions: (1) the power distance index (PDI), (2) the uncertainty avoidance index (UAI), (3) individualism/collectivism (IDV), (4) masculinity/femininity (MAS), (5) long-/short-term orientation (LT/SO) and (6) indulgence/restraint (I/R). The results are now available for 93 countries and regions (www.hofstede-insights.com). Country scores range between 0 and 100. Power distance indicates the degree to which less powerful members of society admit and accept an imbalanced distribution of power. Scores for PDI have a tendency to be higher for Eastern European, Asian, Latin American, and African countries and lower for German-speaking and Western European countries. Uncertainty avoidance indicates the extent of the need for predictability in a society, often reflected by structured, written rules. It tends to be higher in Eastern and Central Europe, in Japan, and in German-speaking countries, and lower in Nordic countries and China. Individualistic cultures, such as rich Western countries, emphasize personal achievement, independence, and self-reliance, resulting in a high level of competition. In contrast, collectivist societies, such as China and Singapore, regard the individual as part of a team and promote close relationships and collaboration among participants. In masculine cultures, such as the USA, the UK, Germany, Hungary, and Japan, the dominant values are achievement and success. Feminine societies, such as the Netherlands and Denmark, are more concerned with caring for others with a cultural emphasis on the quality of life and taking care of others. Long-term-oriented societies focus on perseverance and thrift. A high appreciation for values in this dimension have been historically emphasized in Confucius’ teachings and persist in countries with a Confucian heritage, such as China, Japan, and South Korea. Short-term-oriented nations emphasize respect for traditions, saving face, and personal steadiness and stability. Short-term-oriented countries comprise of the USA, Latin America, Australia, and some African and Muslim countries. Indulgent societies emphasize human desires associated with taking pleasure in life and joy. Restrained societies limit such gratification and regulate them with strict social norms. Indulgent countries can be found among American and Western European countries, while Eastern European and Asian countries tend to have restrained societies (Hofstede, 2011).

### 2.3 The cultural context of the study

**Figure 1:**
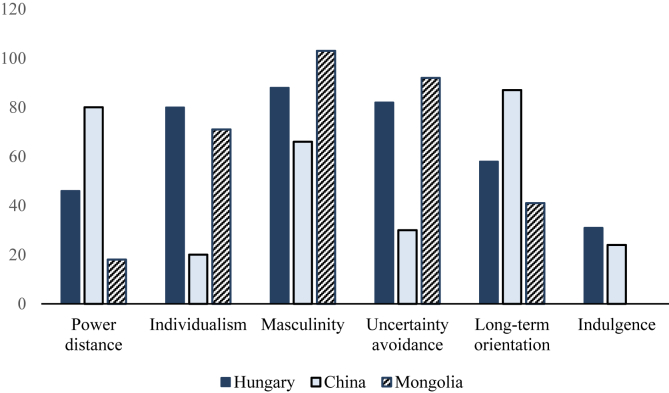
Cultural comparison of Hungary, China and Mongolia through the lens of Hofstede's 6-D model.

The present study aimed to compare survey results through the lens of Hofstede's 6-D cultural types model, in which Hungary is compared to two Asian countries, China and Mongolia. Mongolia is not included in Hofstede's survey: The indices instead were drawn from Rarick et al.’s study (2014). Figure 1 shows the results, with indulgence missing for Mongolia.

As regards the power distance index, the highest is in China. In our comparison, Chinese people are most accepting of inequalities in power among people. Hungary and Mongolia score lower in this dimension, which means that Hungarians and Mongolians tend to believe that the hierarchy is for convenience only, and they more often demand equal rights.

Hungary and Mongolia are highly individualistic societies. Both countries have a strong preference for primarily looking after themselves and their immediate families. In contrast, China has a highly collectivist culture, where members of the society take care of the group more often than themselves.

As regards to the masculinity dimension, in all of these societies, people are driven by success and competition. Students are strongly concerned about examination results as the main criterion for achieving success in life.

Uncertainty avoidance is the lowest in China. This indicates that Chinese people do not feel endangered by ambiguous or unfamiliar situations, and seeking security does not play a central role in their lives. They accept ambiguous or unfamiliar situations. In contrast, Hungary and Mongolia more strongly adhere to rigid rules and structure.

Among the three societies, China is the most long-term-oriented culture, pragmatically adapting traditions when conditions change, and they have high persistence in achieving results. Hungary can be also regarded as long-term-oriented, Mongolia less so.

Finally, both Hungary and China are restrained societies, so both have a tendency toward cynicism and pessimism. They place little emphasis on recreation and manage their desires and impulses (Hofstede, 2001, 2011). Data are missing for Mongolia (Rarick et al., 2014).

#### 2.3.1 The Hungarian context

Hungary is a highly individualistic society, indicating that the social framework is loosely knit and individuals are strongly concerned about themselves and their families and relatively loosely about others. A high score for masculinity suggests that the society is rather driven by achievement, competition and success. Hungarian people also tend to avoid uncertainty, preserve rigid codes of belief and behaviour, and are intrinsically motivated to work hard. Hungarians have a tendency toward cynicism and pessimism and place little emphasis on leisure time.

Education is also characterized by these forces. Hungarian students are mainly concerned with their own success and with admission to university. Competition is high among schools, and students work hard to enter the best institutions. Therefore, school teaching and learning, as well as language teaching and language learning, have long been characterized by memorization, meaning rote learning, which does not necessarily include grasping the meaning of the content. This is changing and there is a tendency now to place emphasis on meaningful learning, with a change towards the use of cognitive learning strategies. Research findings by Doró et al. (2018) indicate that students are increasingly choosing non-rote learning strategies for language learning, such as cognitive or metacognitive strategies.

Research on LLSs does not play a prominent role in language learning research in Hungary. Doró et al. (2018) studied the relationship between language learning and learning characteristics, while Habók and Magyar (2018a) explored the role of LLSs on attitudes towards language learning, language proficiency, and general school achievement. Recently, they reviewed the Strategy Inventory for Language Learning questionnaire and developed a new Self-Regulated Foreign Language Learning Strategy questionnaire. The new instrument is based on Oxford's theory and the theory of self-regulated learning (Habók & Magyar, 2018b, 2019).

#### 2.3.2 The Chinese context

The essence of Chinese culture is characterized by “top to bottom” and “great national unity” (Zhang, 2005). Within this particular cultural context, Chinese people have a strong sense of collectivism (Biggs, 1996). Meanwhile, conceptions of education in China are also significantly influenced and shaped by Confucianism (Lee, 1996; Scollon, 1999), which emphasizes mnemonic and repetitive input in language learning (Wang, 2009) and treats teachers as the authority in the classroom (Wang and Gao, 2008). The result is students are passive learners, tending to “listen to teachers”.

Therefore, the traditional model of education in China is teacher-dominated, and teaching is viewed as the transmission of knowledge (Paine, 1990; Rao, 2006) rather than creating and employing knowledge for immediate objectives (Hu, 2002a, 2002b). In language learning, Chinese students are keener on the traditional learning style related to formal practice, focusing on linguistic accuracy rather than on communicative functions (Wang, 2009; Yu and Wang, 2009). They pay close attention to reception, repetition, review, and reproduction in learning English. Reception refers to students' high receptiveness to teacher-imparted knowledge and knowledge embedded in course books (Paine, 1990). Students engage in intense repetition, which is related to students' reduplicative learning of what is difficult or not understood (Marton et al., 1996), and constant review is considered a crucial factor for successful learning (Wang, 2001). Through constant review and repetition, students are able to rely on existing knowledge and to acquire new knowledge. Accurate reproduction of learned knowledge means students can meticulously reproduce minute language details (Yu, 1984), historically felt to represent the mastery of required knowledge.

However, problems with this traditional English language education in China has led the Ministry of Education of China (MoE of China) to carry out a series of curricular reforms at all levels since 2001 to focus more on learners' comprehensive language competence so as to satisfy contemporary social and economic requirements. Several rounds of reforms have aimed to change an overemphasis on the transmission and interpretation of language knowledge to improve students’ ability to employ their language for real communication. These reforms aim to establish a curriculum that focuses on teaching methods involving students in task oriented activities involving experiential and practical group lessons. Such methods can enhance cooperative learning and help students form positive attitudes by engaging in culturally relevant activities and gaining autonomy in their language learning efforts (cf. MoE of China, 2001, 2011, 2017).

#### 2.3.3 The Mongolian context

Mongolian culture is not well-known; however, during the past two decades, constant political and economic developments have taken place here that have resulted in an extensive inflow of international norms and cultural influences. Dierkes (2012) lists Mongolia as one of eleven countries with significant global economic growth. Rarick et al. (2014) notes that individualism is significantly higher in Mongolia than in the surrounding countries. The Mongolian culture is also rather masculine, suggesting the acceptance of competition and achievement along with the importance of independence and self-reliance. External globalization forces led to requiring Mongolians to require English language in schools. Internal cultural norms require balancing learning methodology with appropriate teaching methods and assessments.

Mongolia has an education system of preschool (Kindergarten), primary school (grades 1–4), lower secondary (grades 5–8), upper secondary (grades 9–10), high school (grades 11–12), vocational training, and higher education. The English language has been taught in Mongolia since 2005 as a major subject in schools and universities. The English language has thus become part of the state-level exams taken by 5th, 9th, and 12th graders in schools and taught as a mandatory course in the first year for two semesters in universities. The Ministry of Education of Mongolia has implemented several programs to improve English language education in Mongolia. Among these are the National English Program (2008–2020; MG, 2008, 293.1) and the “Education 2010–2021” National Program (MG, 2010. 31. 6). These programs were aimed to develop new and improved English language teaching methods and assessments. Under these programs, the Ministry of Education recommended teachers use state-developed core curriculums for all subjects, including English language. These documents were intended to guide teachers on how to teach and how to assess their students (MECS, 2014, 2015).

Efforts to improve English language education in Mongolia are recent and only a few studies have been done yet to assess program effectiveness. While conducting our survey to explore issues in English language learning strategies, we found the following: Altansarnai (2014) mentioned in her study that teaching methods need to be more innovating to develop student learning strategies to help them learn creatively and independently. Altansor (2016) found that students’ age and their developmental characteristics were the most influential factors that require different learning strategies. Even at the university level students learn differently. For example, first-year students learn more by discussing with others, students in their second year appear to learn more with the help of a teacher, third year students learn more by exploring new content from other resources, and students learn more with production practice in their 4th year (Undarmaa and Odgarav, 2017).

Mongolia's success in a global economy requires university students to have good English language skills. Our study showed language learning strategies can help university students improve their English skills. However, more research is needed to ascertain why the current English language teaching methodology has not produced better results in Mongolia compared to fluency in foreign languages in other Asian countries, such as China, Japan, or Vietnam.

#### 2.3.4 Research questions

The following research questions are explored in this paper:

(1) Which types of LLSs are used the most by the different countries?
(2) What are the main differences in learning strategy use among the different countries?
(3) What connections exist between students' overall strategy use and their cultural and educational background?

## 3 Methodology

### 3.1 Sample

A total of 519 university students participated in the survey (Table 1). The Hungarian subsample consisted of 197 Hungarian university students, the Chinese subsample comprised of 200 Chinese university students, and the Mongolian subsample contained 122 Mongolian students. The Hungarian and Chinese subsamples were selected from one university each. The Hungarian subsample was formed of teacher trainees majoring in various disciplines, such as the natural sciences, arts, humanities, and social sciences. The Chinese subsample was made up of humanities and social science students. The Mongolian subsample involved a number of universities in the capital, Ulaanbaatar. The participants were selected from different years and the following fields: the humanities and social sciences, health sciences, law, engineering, economics, and business administration.

**Table 1:** The numbers for the entire sample.

	Hungarian	Chinese	Mongolian	Total
Male	43	171	53	267
Female	154	29	69	252
Total	197	200	122	519

### 3.2 Measurement tool

The Strategy Inventory for Language Learning (SILL, Oxford, 1990) was employed, which is a self-report questionnaire. It is based on Rebecca Oxford's theory and covered two strategy domains: direct (memory, cognitive and compensation) and indirect (metacognitive, affective and social) strategies. Students indicated their responses on a five-point Likert scale ranging from 1 to 5 (never or almost never true of me to always or almost always true of me). The reliability of the questionnaire has been confirmed by numerous studies on language learners from different cultural backgrounds (Oxford and Burry-Stock, 1995). Ganjooei and Rahimi (2008) indicated internal consistency between .86 and .88. Hong-Nam and Leavell (2007) noted high Cronbach's alpha coefficients for their two subsamples (.91 and .94).

### 3.3 Research design and data analysis

Data were collected in three countries, Hungary, China and Mongolia. The questionnaires were administered in online form. The contact person for each university sent the students a link to the measurement tool and wrote a brief accompanying letter on our research objectives. Ethical approval was provided by the IRB at the Doctoral School, University of Szeged. Information on the purpose of the data collection and students' informed consent was obtained in the first part of the measurement tool. It was also noted that students’ responses would be handled confidentially. Students were asked to indicate their participation by clicking on a check box. After deciding to participate in the study, they completed the questionnaires individually in their free time and submitted their responses.

Data analysis was based on descriptive statistics to evaluate students' responses (Cronbach's alpha, mean and standard derivation). We also employed an ANOVA analysis and Dunnett T3 post hoc test to compare the results for the subsamples and individual items. The SPSS statistical package was used for the analysis.

## 4 Results

The Cronbach's alpha coefficients for the SILL questionnaire were examined to analyse internal consistency reliability for each subsample. We found the highest reliabilities for the metacognitive field (Cronbach's alpha = .89–.90) and for the cognitive fields (Cronbach's alpha = .82–.90). The lowest reliability values were registered in the affective (Cronbach's alpha = .55) and memory fields (Cronbach's alpha = .60) in the Hungarian subsample (Table 2).

**Table 2:** Reliability.

Fields	Hungarian	Chinese	Mongolian
Memory	.60	.74	.76
Cognitive	.82	.88	.90
Compensation	.60	.73	.78
Metacognitive	.90	.89	.89
Affective	.55	.78	.82
Social	.80	.84	.83

### 4.1 Overall strategy use and strategy use for the fields on SILL

Our students’ strategy use profile indicated similar LLS use except for two fields. As Table 3 indicates, the strategies preferred by all three subsamples were the metacognitive strategies. Overall, we found nearly the same frequency of strategy use, with no significant differences among the subsamples. There were only two fields in which we could detect significant differences in LLS use. The Hungarian subsample reported significantly higher cognitive strategy use than the Mongolian students, and the Chinese and Mongolian students indicated significantly higher strategy use in the affective dimension. In a comparison of the subsamples, the Hungarian students rated the use of affective strategies the lowest.

**Table 3:** Results for the subsamples in the questionnaire fields.

Fields	Hungarian M(SD)	Chinese M(SD)	Mongolian M(SD)	F(p)	Sig.
Memory	2.95 (.54)	2.98 (.56)	3.03 (.67)	.68 (n.s.)	n.s.
Cognitive	3.14 (.63)	3.10 (.56)	2.92 (.72)	4.84 (< .01)	{1 > 3}
Compensation	3.11 (.62)	3.10 (.58)	3.02 (.78)	.861 (n.s.)	n.s.
Metacognitive	3.28 (.83)	3.16 (.66)	3.28 (.81)	1.65 (n.s.)	n.s.
Affective	2.49 (.62)	3.03 (.65)	3.23 (.77)	55.41 (< .001)	{1 < 2; 1 < 3}
Social	3.08 (.86)	3.12 (.70)	3.06 (.85)	.22 (n.s.)	n.s.
Total	3.01 (.52)	3.08 (.52)	3.09 (.65)	1.14 (n.s.)	n.s.

### 4.2 Individual strategy use

We investigated divergences in individual strategy use in the relevant fields to discover further differences. The results for strategy use in the cognitive field in the Hungarian and Mongolian subsamples are summarized in Table 4.

**Table 4:** Results for individual strategy use in the cognitive field in the Hungarian and Mongolian subsamples.

Strategy	Hungarian M(SD)	Mongolian M(SD)	F(p)	Dunnett T3 (p)
10. I say or write new English words several times.	3.83 (1.04)	3.29 (.97)	15.35 (< .001)	(.023)
11. I try to talk like native English speakers.	3.32 (1.16)	2.92 (1.20)	4.67 (.01)	(.013)
15. I watch English language TV shows in English or see movies in English.	3.59 (1.24)	3.16 (1.11)	8.57 (< .001)	(.004)
20. I try to find patterns in English.	3.79 (1.00)	2.97 (1.01)	46.45 (< .001)	(< .001)
22. I try not to translate word for word.	3.68 (1.11)	3.11 (1.11)	21.09 (< .001)	(< .001)
23. I make summaries of information that I hear or read in English.	3.37 (.99)	2.94 (1.16)	11.09 (< .001)	(.003)

Fourteen strategies were identified in the cognitive field, and six of these (shown in Table 4) were significantly different between Hungary and Mongolia. The other field where significant differences occurred among the countries was the affective field. There were six statements related to this field. Table 5 summarizes the differences among the subsamples.

**Table 5:** Results for individual strategy use in the affective field in the subsamples.

Strategy	Hungarian M(SD)	Chinese M(SD)	Mongolian M(SD)	F(p)	Dunnett T3 (p)
39. I try to relax whenever I feel afraid of using English.	3.16 (1.14)	3.19 (.88)	3.47 (1.01)	3.82 (.02)	{3 > 1}(.023) {3 > 2}(.039)
40. I encourage myself to speak English even when I am afraid of making a mistake.	3.58 (1.12)	3.13 (.92)	3.38 (1.09)	9.14 (< .01)	{1 > 2}(< .001)
4l. I give myself a reward or treat when I do well in English.	2.36 (1.26)	3.05 (1.00)	3.56 (1.28)	46.53 (< .001)	{2 > 1}(< .001) {3 > 1}(< .001) {3 > 2}(< .001)
42. I notice if I am tense or nervous when I am studying or using English.	2.79 (1.33)	3.08 (.86)	3.52 (1.07)	15.81 (< .001)	{2 > 1} (< .035) {3 > 1}(< .001) {3 > 2}(< .001)
43. I write down my feelings in a language learning diary.	1.11 (.39)	2.78 (1.05)	2.55 (1.15)	192.74 (< .001)	{2 > 1} (< .001) {3 > 1}(< .001)
44. I talk to someone else about how I feel when I am learning English.	1.89 (1.08)	2.97 (.96)	2.93 (1.02)	65.41 (< .001)	{2 > 1} (< .001) {3 > 1}(< .001)

## 5 Discussion

This research contributes to FL acquisition in two ways. First, we systematically analysed LLS use among Hungarian, Chinese and Mongolian university students, resulting from subsamples from different cultural and linguistic backgrounds. Second, we compared university students' LLS use and examined the relations between them to identify culture-specific similarities or differences among the samples. We employed Hofstede's 6-D model of cultural values to explain cultural divergences.

The results showed a relatively medium frequency of overall use in each of the participating subsamples, thus supporting the importance of cultural background. In all of these participating subsamples, English is learnt as a foreign language, with EFL taking place in a setting where English is not the primary means of communication. Therefore, students are not strongly motivated to learn English and use a great variety of LLSs because they mainly practise English in the FL classroom, but not in their daily lives.

The findings also showed that the Hungarian, Chinese, and Mongolian university students employ metacognitive LLSs the most frequently. For Hungarian students, these results are in line with those of our previous study with a purely Hungarian sample (Habók & Magyar, 2018a, 2020). Hungarian students prefer strategies that set clear goals for consciously improving English knowledge. They learn for success and monitor their progress. They also think about their own EFL achievement, which is their most important focus. This may be rooted in Hungarian culture being a rather individualistic and masculine-oriented society. Hungarian students learn languages to be admitted to university, to find a good job, or for other aims, but they most often learn for success and career (Habók & Magyar, 2018a, 2020). The second most used LLS was the cognitive strategy type for the Hungarian subsample. They often “try to find patterns in English”, “not to translate word for word” and “say or write new English words several times”. These LLS choices are also conducive to lifelong learning and closely linked to a success orientation.

Among the metacognitive strategies, the most characteristic strategy for the Chinese participants was that they “pay attention when someone speaks English” and seek out people with whom to speak a FL. They also prefer social strategies, such as learning together, cooperating and helping each other. These may originate in Chinese society being a collectivist culture, where other people or the community are the focus. The Chinese results are in line with those of Rao (2006) and Zhang and Xiao (2006), who also found a frequent use of these types of metacognitive strategies. Similarly, Wu (2008) pointed out that the regular use of metacognitive and social strategies is a characteristic of more proficient university students.

The Mongolian students’ pattern of metacognitive strategy use is rather similar to that of Hungarian students; they also set clear goals to improve their English and are driven by success, which is a main characteristic feature of masculine societies. They also think a great deal about their own development and prefer affective strategies that are self-focused, such as giving themselves a reward or considering their feelings connected to language learning. These features may be tied to individualism.

Comparing the different subsamples of the three countries, we identified significant differences in three cases. First, the Hungarian subsample reported significantly higher cognitive strategy use than the Mongolian students. Specifically, Hungarian students tend to “say or write new English words”, “try to talk like native speakers”, “watch English language TV shows”, “try to find patterns in English”, “try not to translate word for word”, and “make summaries of information that I … read in English” more than to the extent that Mongolian students do. These differences are unique in our cultural comparison because they could not be linked to our cultural dimensions. Second, the affective dimension was significantly lower in the Hungarian subsample. Hungarian students do not relax, do not “give [themselves] a reward or treat when [they] do well in English”, do not notice when they are nervous, and do not write down or speak about their feelings to other people. These results imply that Hungarian responders do not like to express their emotions, cannot relax and do not celebrate their success. This feature is related to the indulgence dimension, in which Hungary demonstrates a rather low score.

## 6 Limitations

We must also note some limitations of our research. We used a self-report questionnaire, and this was the single instrument for our data collection. Administering the questionnaire could be supplemented with tests to determine students' proficiency level or quantitative methods involving a think-aloud procedure could be used to discover students’ views of LLSs. Another limitation is that the number of participants was low and the participating students came from different disciplines. In a later study, it will be possible to expand the sample number and involve other universities to make the sample more representative.

## 7 Conclusion and pedagogical implications

Our questionnaire-based study showed that the participating students used metacognitive strategies the most among their FL learning strategies. Obviously, not all strategies can be used at once, and there are results that highlighted that effective learners use a small number of strategies depending on the learning task, but it is important that students learn and practise all strategy types, especially during the first phase of language education. A broad knowledge of strategies enables them to choose those appropriate to the learning task. Our study reinforced some previous research, which stated that learning strategy use is strongly determined by other factors, such as cultural and educational background. The learning process is shaped by particular cultural and pedagogical backgrounds through classroom activities and preferred teaching methods. Therefore, in the EFL classroom, the language learning process, strategy use and cultural influences cannot be separated; rather they are interwoven.

## Declarations

### Author contribution statement

Anita Habók, Andrea Magyar: Conceived and designed the experiments; Performed the experiments; Analyzed and interpreted the data; Contributed reagents, materials, analysis tools or data; Wrote the paper.

Yunjun Kong, Jargaltuya Ragchaa: Performed the experiments; Analyzed and interpreted the data; Contributed reagents, materials, analysis tools or data; Wrote the paper.

### Funding statement

This work was supported by the University of Szeged Open Access Fund (Grant number: 5001).

### Data availability statement

The data that has been used is confidential.

### Declaration of interests statement

The authors declare no conflict of interest.

### Additional information

No additional information is available for this paper.
